# Supplementary material for: Dance to Prosper: Benefits of Chinese Square Dance in QOL and the Moderating Roles of Aging Stereotypes
Source: Int J Environ Res Public Health. 2022 Dec 8;19(24):16477. doi: 10.3390/ijerph192416477 (PMC9778596; doi:10.3390/ijerph192416477)
Supplement: Supplementary file 1 [file ijerph-19-16477-s001.zip › ijerph-1962607-supplementary.pdf]

Supplemental Online Materials for Dance to Prosper: The Benefits of Chinese Square Dance and the Moderating Roles of Aging Stereotypes

Measures

Demographics

Participants reported their age, gender, height and weight, family income, marital status, education, and features of their own dancing experience. Although some factors about the dancing features were expected to be potential predictors, the preliminary analysis revealed that these features did not contribute to ATOA and QoL change. Therefore, most of the demographic characteristics were not included in the final model. However, to understand the inner structure of the dancing group and to portray a typical profile for Chinese square dancers, such data were of great importance. **Table S1** displays the information of participants in this research, which represents a typical sample among the 100 million dance lovers.

Activity volume of Chinese square dance

According to research on activities [1], the time engaging in an activity was allowed to be transformed into categorical variables to facilitate analysis and explanation. In this method, the dance duration of our participants was labeled from "low engagement" to "high engagement"according to their total number of minutes spent for a week. The time range and splitting points for Chinese square dance engagement are presented in **Table S2.** In the sample, participants had a high dance participation rate, and "0 engagement" participants were less than 30%. Additionally, the researchers did not expect no participation and low participation to cause a distinct difference according to the number of minutes people spent at the low intensity level. Therefore, "0" was also included in the low-engagement group, leaving three groups to compare in the final analysis.

Results

Moderated mediation

The conditional indirect effects of dancing volume on QoL through ATOA at varying levels of negative and positive IOA (1 SD above the mean and 1 SD below the mean) were tested using MPLUS 8.3 version. The results separately show relative indirect effects for the two dummy groups. The dance volume × negative IOA interactions were significant for the two dummy variables: both Dummy 1 and Dummy 2 predicted ATOA at high levels of negative IOA. Furthermore, significant conditional indirect effects (moderated mediation) on QoL via ATOA for Dummy 1 and Dummy 2 were tested. Results for the specific coefficients and figures for simple slopes are reported in the main document, and the structural equation model with standardized parameter estimates is presented here in the Supplementary Material in Figure S1.

As for model with positive aging stereotype, the results revealed a nonsignificant Dummy 1 × positive IOA interaction on ATOA and a marginal significant Dummy 2 × positive IOA interaction, as presented in **Table S3**. **Figure S3** plots the simple slopes of dance duration on ATOA at various values of positive IOA (mean, ±*SD*). The interactions on ATOA indicate that the effect of dance duration depends less on the degree of positive IOA. Only when people had enough difference in dance duration did the effect of positive IOA differences appear apparent. Similarly, the conditional indirect effect (moderated mediation) on QoL via ATOA is only significant for Dummy 2, and the details of coefficients are presented in **Table S4.** An overall structural equation model with standardized parameter estimates is presented in **Figure S2.**

Table S1. The overall demographic profile of the dancers.

Variable	M (SD) or *n* (%)
**Personal factors**	
Age	48.83 (9.57)
Income per year	89223.5 (78221.6)
BMI	26.33 (8.74)
Urban	356 (76.4%)
Rural	84 (23.6%)
**Gender**	
Female	371 (84.1%)
Male	70 (15.9%)
**Marital status**	
Married	405 (91.8%)
Not married/other situation	35 (8%)
**Experience in dancing**	
<one month	14 (3.5%)
1–3 months	31 (7.7%)
3months–6 months	45 (11.2%)
6 months–one year	92 (22.8%)
More than one year	221 (54.8%)
**Education**	
< Junior high school	12 (2.7%)
Junior high school	89 (20.2%)
High school	139 (31.6%)
Associate degree	90 (20.5)
Bachelor's	109 (24.8%)
Graduate degree	1 (0.2%)
**Team size**	
<10	24 (5.4%)
10–30	185 (42%)
30–50	108 (24.5%)
>50	86 (19.5%)
**missing**	38 (8.6%)

Note: The table presents distributions as percentages for factors and means for continuous variables.

Table S2. Groups of Chinese square dancers by activity duration according to the time range.

Groups by duration	Low	Medium	High
**Weekly engagement (minutes)**	0–410	410–600	600–2160

Note: Times performing Chinese square dance were counted and summed for seven days. After integration, the sample was evenly divided into three groups according to tertiles of the time range.

Table S3. Regression results on ATOA in model with positive image of aging.

	B	***SE***	***t***	***p***
Predictor				
Constant	12.78	0.94	13.66	<0.001
Age (covariate)	−0.001	0.01	−0.09	0.93
Gender (covariate)	0.14	0.24	0.57	0.56
BMI (covariate)	−0.03	0.03	−1.02	0.31
Positive Image of Aging	0.69	0.17	4.12	<0.001
Medium dance volume (Dummy 1)	<0.001	0.22	0.00	1.00
High dance volume (Dummy 2)	0.40	0.20	1.99	0.05
Dummy 1 ×Positive Image of Aging	−0.29	0.25	−1.15	0.25
Dummy 2 ×Positive Image of Aging	−0.43	0.22	−1.91	**0.05**
Conditional Effect of Medium dance volume (D1) on ATOA				
at values of Positive image of aging				
Positive image of aging				
−1 *SD*	0.285	0.364	0.781	0.435
+1 *SD*	−0.285	0.292	−0.973	0.330
Conditional Effect of High dance volume (D2) on ATOA				
at values of Positive image of aging				
Positive image of aging				
−1 *SD*	0.825	0.326	2.532	**0.011**
+1 *SD*	−0.028	0.271	−0.104	0.917

Note. Unstandardized regression coefficients are reported.

Table S4. Regression results on QoL in model with positive image of aging.

	B	*SE*	*t*	*p*
Predictor				
Age (covariate)	0.20	0.06	3.29	<0.001
Gender (covariate)	−1.01	1.41	-0.72	0.47
BMI (covariate)	−0.15	0.22	-0.69	0.49
Medium dance volume (Dummy 1)	3.51	1.48	2.38	0.02
High dance volume (Dummy 2)	3.31	1.50	2.21	0.03
ATOA	2.26	0.46	4.96	<0.001
Conditional Indirect Effect of Medium dance volume (D1) on QoL via ATOA at values of Positive image of aging				
Positive image of aging				
−1 *SD*	0.644	0.830	0.776	0.438
+1 *SD*	−0.645	0.703	-0.917	0.359
Conditional Indirect Effect of Medium dance volume (D2) on QoL via ATOA at values of Positive image of aging				
Positive image of aging				
−1 *SD*	1.867	0.824	2.267	0.023
+1 *SD*	−0.064	0.625	-0.103	0.918

Note. Unstandardized regression coefficients are reported.

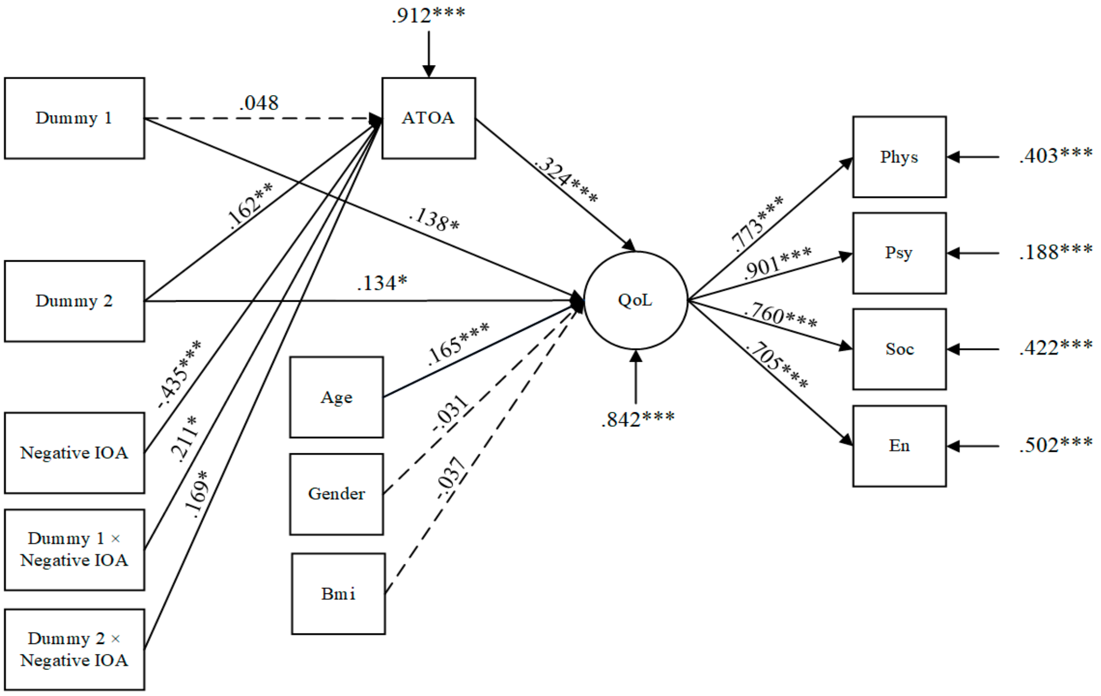

Figure S1. Structural model for QoL with negative IOA as the moderator. Note. The circles represent latent variables, and rectangles are observed variables. The broken lines indicate effects that are not statistically significant. Path coefficients are standardized MPLUS parameter estimates. The subdomains of QoL scales are: Phys = physical, Psy = psychological, Soc = social, En = environmental. * $p < 0.05$; ** $p < 0.01$; *** $p < 0.001$.

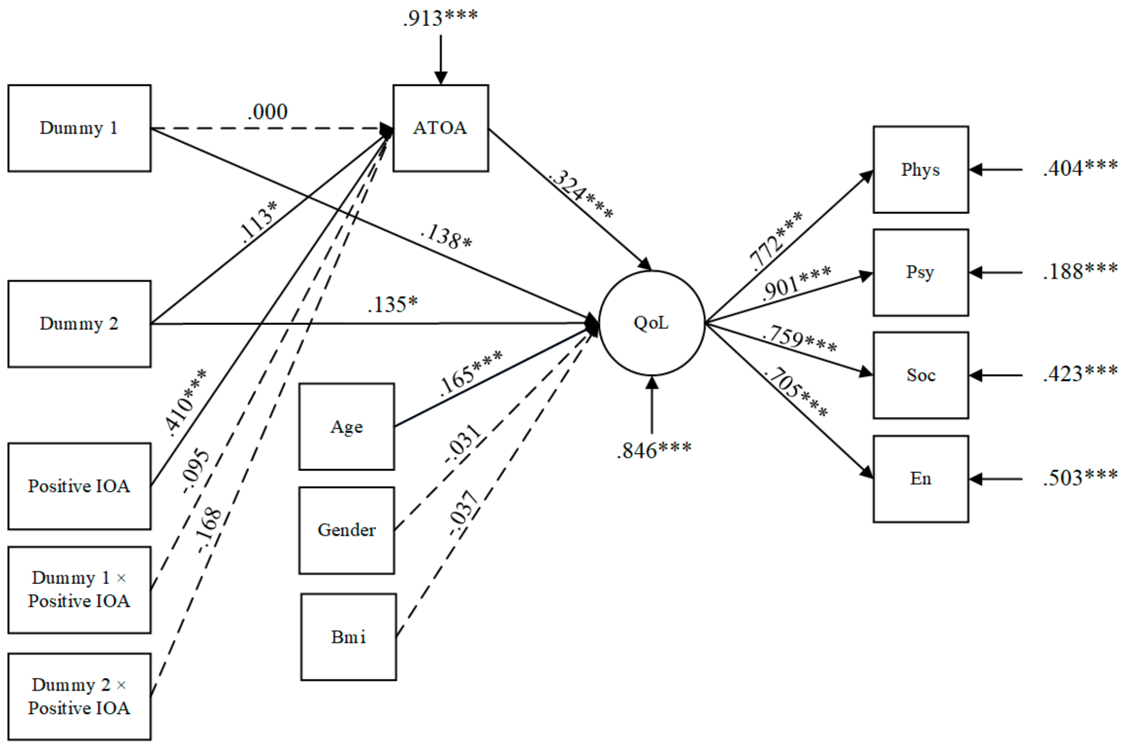

Figure S2. Structural model for QoL with positive IOA as the moderator. Note. The circles represent latent variables, and rectangles are observed variables. The broken lines indicate effects that are not statistically significant. Path coefficients are standardized MPLUS parameter estimates. The subdomains of QoL scales are: Phys = physical, Psy = psychological, Soc = social, En = environmental. * $p < 0.05$; *** $p < 0.001$.

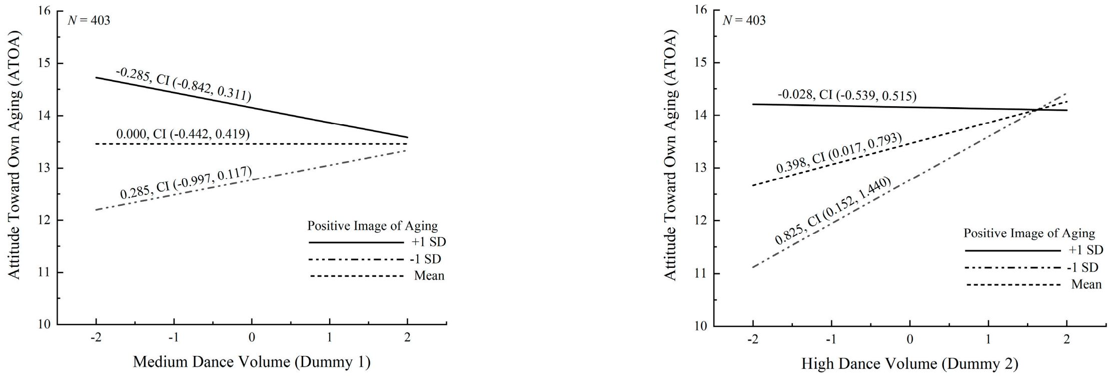

(**a**) (**b**)

Figure S3. Conditional effects (simple slopes) of dance duration on ATOA at various values of positive IOA (mean, plus, and minus one standard deviation from the mean) for Dummy 1 (**a**) and for Dummy 2 (**b**).

Supplemental References

1. Morrow-Howell, N., Putnam, M., Lee, Y. S., Greenfield, J. C., Inoue, M., & Chen, H. (2014). An Investigation of Activity Profiles of Older Adults. *The Journals of Gerontology Series B: Psychological Sciences and Social Sciences*, *69*(5), 809–821.
